# Application of ensemble methods to analyse the decline of organochlorine pesticides in relation to the interactions between age, gender and time

**DOI:** 10.1371/journal.pone.0223956

**Published:** 2019-11-13

**Authors:** Aleysha Thomas, Nicole M. White, Leisa-Maree Leontjew Toms, Kerrie Mengersen

**Affiliations:** 1 ARC Centre of Excellence for Mathematical and Statistical Frontiers, Queensland University of Technology, Brisbane, Queensland, Australia; 2 School of Public Health and Social Work, Queensland University of Technology, Brisbane, Queensland, Australia; University of Florida, UNITED STATES

## Abstract

Organochlorine pesticides (OCPs) are toxic chemicals that persist in human tissue. Short and long term exposure to OCPs have been shown to have adverse effects on human health. This motivates studies into the concentrations of pesticides in humans. However these studies typically emphasise the analysis of the main effects of age group, gender and time of sample collection. The interactions between main effects can distinguish variation in OCP concentration such as the difference in concentrations between genders of the same age group as well as age groups over time. These are less studied but may be equally or more important in understanding effects of OCPs in a population. The aim of this study was to identify interactions relevant to understanding OCP concentrations and utilise them appropriately in models. We propose a two stage analysis comprising of boosted regression trees (BRTs) and hierarchical modelling to study OCP concentrations. BRTs are used to discover influential interactions between age group, gender and time of sampling. Hierarchical models are then employed to test and infer the effect of the interactions on OCP concentrations. Results of our analysis show that the best fitting model of an interaction effect varied between OCPs. The interaction between age group and gender was most influential for hexachlorobenzene (HCB) concentrations. There was strong evidence of an interaction effect between age group and time for *β*-hexachlorocyclohexane (*β*-HCH) concentrations in >60 year olds as well as an interaction effect between age group and gender for HCB concentrations for adults aged >45 years. This study highlights the need to consider appropriate interaction effects in the analysis of OCP concentrations and provides further insight into the interplay of main effects on OCP concentration trends.

## Introduction

Organochlorine pesticides (OCPs) are a class of lipophilic persistent organic pollutants (POPs) that exist in the environment and accumulate in human tissue [1–4]. POPs are subject to long range transport via air, water and dust [4, 5] and the concentrations of POPs are known to degrade slowly in humans [6, 7]. In Australia, pesticides including dichlorodiphenyltrichloroethane (DDT), hexachlorocyclohexanes (HCHs) and hexachlorobenzene (HCB) were routinely used for agricultural applications prior to their deregistration in 1985 [8, 9]. Short and long-term exposure to POPs, including OCPs, has been associated with negative health impacts such as cancers and lymphoma, as well as reproductive and neurological effects [10, 11]. These adverse effects have motivated epidemiological studies into POP concentrations in blood serum [12–14], other tissues [15, 16], and changes in concentrations over time [17–22].

The individual effects of age, gender and time of sample collection on POP concentrations are a widely studied subject of monitoring reports. In studies conducted on serum and breast milk samples collected in the late 1990’s and early 2000’s (after the production and use of the POPs) researchers around the world, including in Australia [17, 18], have identified that POP concentrations are higher in older adults [17, 23–25], vary between genders [17, 26, 27] and generally decrease over time [17–20]. This paper analyses five OCP concentrations sampled from children and adults in Australia between 2002 and 2012 [22]. Beyond individual effects, there are gaps in our understanding of how the interactions between age, gender and time affect the levels of POP concentrations in a population [21]. For example, an interaction between age group and gender would indicate differing concentrations between men and women of the same age group. Similarly, an interaction between age group and time of sample collection would suggest non-parallel age-specific trends in OCP concentrations over time and provide insight into these trends as the population ages. Studies in the field have not yet distinguished the effects of such interactions on the trend of OCP concentrations [21].

Interactions between age group, gender and time of sample collection on long term OCP concentrations have previously been statistically evaluated by Thomas et al. [22]. Similar data has been studied by Woodruff et al [28]. Using analysis of variance (ANOVA) and multiple comparisons, these authors identified significant interactions between age group and gender as well as time of sample collection and gender. Other studies have used similar methods to identify trends in OCP concentrations but did not investigate interaction effects [17, 18].

The paper proposes a two-stage statistical model for the discovery of influential interaction effects between the key covariates age group, gender and time. In the first stage of the analysis, boosted regression trees (BRTs) are applied to elucidate interactions for each OCP independently. Resulting interactions are incorporated into an hierarchical model for describing temporal trends in OCP concentrations as a function of the interactions. BRTs are a flexible, data-driven method to identify interactions that affect the response variable. Hierarchical models are a generalisation of linear models where parameters can vary at more than one level. The proposed hierarchical model in this study is formulated within the Bayesian framework so that a range of prior distributions can be used to incorporate the structure of the data into the model.

## Materials and methods

This section gives a brief summary of the data analysed followed by a description of each model of the two stage analysis namely, BRTs and hierarchical models. The methods adopted to evaluate the performance of the models are briefly summarised here.

### Data

The two stage analysis was applied to concentrations of HCB, *β*-HCH, trans-nonachlor, *p,p*’-DDE and *p,p*’-DDT measured from pooled samples of de-identified surplus human blood serum from males and females collected in Brisbane, Australia in 2002/03, 2006/07, 2008/09, 2010/11 and 2012/13 across the age groups 5-15, 16-30, 31-45, 46-60 and >60 years. The age group 0-4 years was available but excluded from the analysis due to missing data for the samples collected in 2002/03.

Each pool consisted of 100 individual serum samples for 2002/03, 2008/09, 2010/11 and 2012/13. Pooled samples collected in 2006/07 comprised up to 30 samples per pool. A total of 12,175 individuals made up of 183 pools. There are more pools for children aged 5-15 years in 2006/07 as these samples were originally collected for a different study as pools for the age groups 5-9, 9-12 ad 12-15 years [29].

OCP concentrations in serum are broadly representative of a person’s body burden of OCPs. There are factors which can affect an individual’s body burden such as exposure to OCP through occupation or lifestyle as well as factors affecting elimination such as breastfeeding or lipid mobilization as occurs during weight gain and loss [30]. An advantage of using pooled samples is that is has the effect of diluting any high or low concentrations to estimate the ‘average’ OCP concentration in the pool and eliminate variability.

Further details on data collection are provided in the Supporting Information (S1 File) and a summary of the data are provided in Thomas et al. [22]. The number of pools sampled at each time for each gender and age group is summarised in Table 1 below.

**Table 1:** Summary of the total number of pooled sample of OCPs, by each level of time of sample collection, gender and age group.

		Time of sample collection				
Age Group	Gender	2002/03	2006/07	2008/09	2010/11	2012/13
**5-15**	**Male**	3	15	2	4	2
	**Female**	3	17	2	4	2
**16-30**	**Male**	3	2	2	4	2
	**Female**	3	2	2	4	2
**31-45**	**Male**	1	2	2	4	2
	**Female**	1	2	2	4	2
**46-60**	**Male**	3	2	2	4	2
	**Female**	3	2	2	4	2
**>60**	**Male**	3	2	2	4	2
	**Female**	3	2	2	4	2
**Total**		26	48	20	40	20

Ethics approval for this study was granted by The University of Queensland (UQ) Medical Research Ethics Committee and Queensland University of Technology (QUT) Ethics Committee (Ethics Approval Number: 2013000317). As per the ethics approval, the samples were de-identified and pooled therefore no consent was required from the participants.

### Boosted regression trees

Boosted regression trees (BRTs) offer a non-parametric approach to predictive modelling by combining Classification and Regression trees (CART) [31] with a boosting algorithm [32] to refine model predictions. A CART is a decision tree that determines covariate-based rules to recursively split the data into subgroups of observations with a similar response [33]. Boosting is a stage wise process whereby multiple simple models are combined to enhance predictive performance [34]. In a BRT, the loss in predictive performance is represented by a loss function. For continuous responses, changes in deviance is a commonly chosen loss function [35]. The initial regression tree has the lowest possible loss function. Using the boosting algorithm, regression trees are sequentially applied to the residuals of the previous tree until there is no further improvement in predictive performance [36].

BRTs were adopted in this paper to determine the relative importance of age group, gender and time, and their interaction, for predicting OCP concentrations. For the data described in the earlier section, BRTs were applied separately to concentration measurements for HCB, *β*-HCH, trans-nonachlor, *p,p’*-DDT and *p,p’*-DDE. For the first inference, BRTs were used to determine the relative importance of predictor variables and possible interactions for each OCP.

The relative importance of a predictor variable in each BRT was calculated by weighting the number of times the variable was selected for a split by the squared improvement to the model caused by the split [36]. This improvement was determined by the least squares improvement criterion described in Friedman et al. [37]. The weighted value was averaged over all trees and scaled so that the sum of the relative importance of all predictor variables added up to 100 [36]. On this scale, larger values indicated stronger influence of the predictor variable on the response. The partial dependence plot of the categorical variables on the response illustrates the marginal effect of each fitted factor; a marginal effect greater than zero indicates higher concentrations than expected and values below zero indicate lower concentrations.

To evaluate the relative importance of interaction effects, each two-way interaction between predictor variables is related to BRT predictions using a linear model [36]. The residual variance of the linear model is then used as a measure of relative strength of the interaction fitted by the BRT [36].

All BRT models were fit in R v3.3.2 using the *gbm* package and *brt* extension [35, 36] with 50% of the data used for each training and test set. Results of the BRT analysis are detailed in the Results section. Important interactions identified by BRTs motivated the development of models for the second stage of the analysis, the main results of the BRT analysis are also referenced in the next section.

### Hierarchical model

BRTs are well suited for modelling the influence of predictors and possible interactions but cannot be used to test differences between sub-groups or assess the effect sizes. Therefore, hierarchical models were used to infer the effects of predictors and interactions on OCP concentrations. Hierarchical linear models expand upon the linear modelling framework for the analysis of nested data structures [38].

Three hierarchical models are outlined in this section, labelled Models 1-3. Each model assumed a common response variable, *y*_*agt*_, defined as the observed OCP concentration for age group *a* (*a* = 1, …, 5), gender *g* (*g* = 1, 2) and time *t* (*t* = 1, …, 5). To reduce notation, each model is defined for a single OCP.

Model 1 was motivated by the interaction between age group and time, which was identified as the most influential interaction for *β*-HCH, Trans-nonachlor, *p,p’*-DDE and *p,p’*-DDT. This model took the following form for each *y*_*agt*_:

$$\begin{array}{cc} y_{agt} & ∼N(\beta_{at},\sigma^{2})\\ \alpha_{g} & ∼N(0,10000)\\ \beta_{at} & ∼N(\beta_{a(t-1)},\omega^{2})\\ \beta_{a0} & ∼N(0,\omega^{2})\\ \sigma & ∼U(0,100)\\ \omega & ∼U(0,100) \end{array}$$ (1)

where the random effect, *β*_*at*_, modelled the interaction between age group and time. These effects were assumed to be temporally correlated, such that *β*_*a*0_, …, *β*_*at*_ was the predicted temporal trend in concentrations for age group *a*. The choice of hyperparameter values for *α*_*g*_, *σ* and *ω* were chosen to indicate a lack of prior knowledge about the nature of the relationships between the response and explanatory variables. Differences between gender were not included in this hierarchical model. Hence, Model 1 specified that the changes in OCP concentrations over time were the same for males and females but began at different concentrations depending on the age group.

For HCB concentrations, the BRT analysis revealed a key interaction between age group and gender. This finding motivated a second hierarchical model to account for this interaction, labelled Model 2 and defined in Eq (2).

$$\begin{array}{cc} y_{agt} & ∼N(\alpha_{ag},\sigma^{2})\\ \alpha_{ag} & ∼N(\delta_{a},\phi^{2})\\ \delta_{a} & ∼N(0,10000)\\ \phi & ∼U(0,100) \end{array}$$ (2)

In Model 2, the random effect, *α*_*ag*_ was included to account for interactions between age group and gender. An intercept term for each age group, *δ*_*a*_, was also included. Model 2 specifies that the changes in OCP concentrations can be described by the differences between age groups and genders and are not affected by the time.

Model 3 accounted for all interactions with age group, therefore combining BRT findings across all OCPs:

$$\begin{array}{cc} y_{agt} & ∼N(\alpha_{ag}+\beta_{at},\sigma^{2})\\ \alpha_{ag} & ∼N(\delta_{g},\phi^{2})\\ \beta_{at} & ∼N(\beta_{a(t-1)},\omega^{2})\\ \beta_{a0} & ∼N(0,\omega^{2}) \end{array}$$ (3)

where each observation is described by random effects *α*_*ag*_ and *β*_*at*_ and an overall variance. *α*_*ag*_ was defined by an overall effect of each gender. The definition of *β*_*at*_ was as per Model 1.

Note that in Model 2, *α*_*ag*_ was described by a normal distribution with a mean of *δ*_*a*_ and an overall variance, whereas in Model 3, it was described by a normal distribution with a mean of *δ*_*g*_ and an overall variance. As in Model 1, the priors for *β*_*a*0_, *δ*_*a*_ and *δ*_*g*_ were non-informative normal distributions with a mean of zero and large variance. All variances were also given non-informative priors.

The second inference focused on the interaction effects on OCP concentrations. The posterior means and 95% credible intervals of the OCP concentrations were calculated from the posterior distribution of the parameter estimates to compare interactions and visualise the temporal trends.

The third inference summarised the changes in OCP concentrations over time. The percent change and 95% credible interval of OCP concentrations between the years of sample collection was calculated from the posterior distribution of parameter estimates generated by the hierarchical model. For an overall view of the change, the percent change in OCP concentrations between 2002/03 and 2012/13 was calculated. The change between each year of sample collection was also calculated to distinguish any specific trend. To evaluate cohort effects, estimated OCP concentrations for each age group in 2002/03 were compared with their extrapolated age group in 2012/13.

The proposed hierarchical models were estimated by Markov Chain Monte Carlo (MCMC) using ‘JAGS’ in R version v3.3.2 [39–41]. Parameter estimates were based on two MCMC chains each comprising 10,000 iterations following a burn-in period of 10,000 iterations. The MCMC chains reached convergence, as assessed by the Gelman-Rubin diagnostic (S1 Fig).

### Model assessment

The BRT model fit was graphically evaluated by comparing the observed OCP concentrations to the model’s predicted OCP concentrations. In a model with a good fit, the difference between the observed and predicted OCP concentrations would be small. On a graph with identical axes, the plotted values would be close to or on the diagonal line; larger differences would be plotted farther from the diagonal line.

The hierarchical models were assessed with two approaches, namely the predictive intervals (PI) [42] and deviance information criterion (DIC) [43].

For each hierarchical model, the (1 − *α*)% predictive intervals (PI) were determined for each observation, *y*_*i*_. In the study, three significance levels were considered, namely *α* = 0.2, 0.1 and 0.05. The proportion of observed values that fell into the respective PIs was calculated. For an adequate model, approximately (1 − *α*) of the observations should fall in their respective intervals [42].

The DIC [43] for each hierarchical model based on data *y* with parameters *θ* has the following form,

$$\begin{array}{c} DIC=D(\theta^{*})+2pD \end{array}$$ (4)

where *D*(θ*) is a measure of the goodness of fit of the model as represented by the posterior mean deviance, given an estimate of *θ*,

$$\begin{array}{c} D\left(\theta^{*}\right)=-2logp\left(y|\theta^{*}\right) \end{array}$$ (5)

and *pD* is the complexity of model measured by an estimate of the effective number of parameters defined by the difference between the average deviance and the deviance given an estimate of the model parameters,

$$\begin{array}{c} pD=\bar{D(\theta )}-D\left(\theta^{*}\right) \end{array}$$ (6)

All models were also evaluated with root mean squared error (RMSE). RMSE is a measure of the differences between observed values and predicted values from a model. This measurement of goodness of fit was calculated by Eq 7.

$$\begin{array}{c} RMSE=\sqrt{\frac{1}{n}\sum_{j=1}^{n}{\left(y_{j}-\hat{y_{j}}\right)}^{2}} \end{array}$$ (7)

where, in a sample of size *n*, *y*_*j*_ and $\hat{y_{j}}$ refer to observed and predicted values, respectively, for values *j* = 1, …, *n*.

### Sensitivity assessment

Given the discrepancy in the number of samples for the 5-15 age group sampled in 2006/07 (Table 1), it was possible that the unbalanced nature of the data could distort the modelling results of the analysis. The sensitivity of the models with respect to the number of samples was therefore assessed as follows. Four pooled samples of the 5-15 age group in 2006/07 were randomly selected and analysed with the remaining age groups under Models 1, 2 and 3. Subsequent parameter estimates were compared with estimates obtained using the original number of samples.

## Results

### Model assessment

#### BRTs

**Fig 1:**
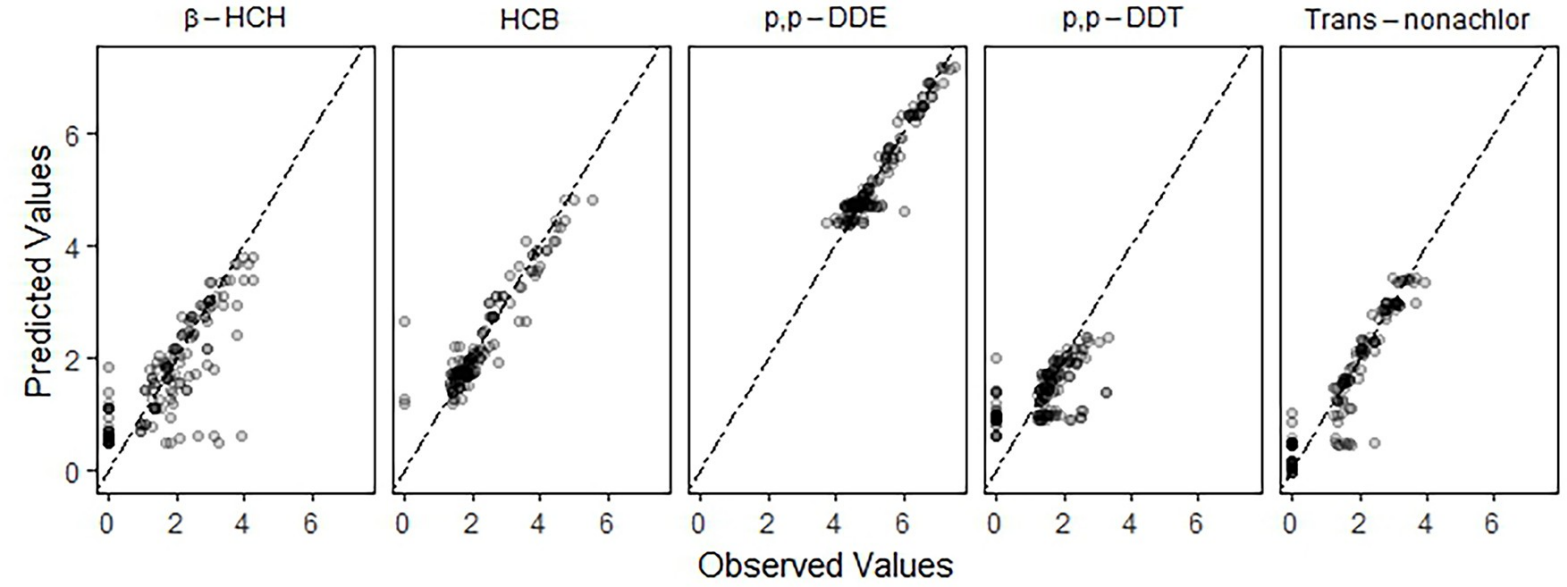
Graphical evaluation of BRT models for OCP concentrations. Graphical comparison of observed (horizontal axis) and predicted (vertical axis) log-transformed OCP concentrations to illustrate accuracy of BRT models for HCB, *β*-HCH, Trans-nonachlor, *p,p’*-DDE and *p,p’*-DDT concentrations. BRT models for HCB and *p,p’*-DDE had more accurate predictions than models for *β*-HCH, Trans-nonachlor and *p,p’*-DDT. All points in these plots have partial transparency so that a darker shade is indicative of overlapping points. Unit of OCP concentrations were in ng/g lipid.

Model assessment demonstrated larger RMSE values for *β*-HCH and *p,p*’-DDT concentrations compared to other OCPs, indicative of larger residuals for the former (Table 2). Graphical evaluation of the observed and predicted BRTs values indicated an acceptable goodness of fit for all OCPs (Fig 1). The predictions of *β*-HCH and *p,p*’-DDT concentrations from the BRT models were less accurate than those of HCB, Trans-nonachlor and *p,p*’-DDE concentrations.

**Table 2:** RMSE values for BRT models of HCB, *β*-HCH, Trans-nonachlor, *p,p*’-DDE and *p,p*’-DDT.

OCP	RMSE
HCB	0.35
*β*-HCH	0.71
Trans-nonachlor	0.40
*p,p*’-DDE	0.24
*p,p*’-DDT	0.66

#### Hierarchical model

The best fitting model varied between OCPs (Table 3). The PIs obtained for Models 2 and 3 for Trans-nonachlor, *p,p*’-DDE and *p,p*’-DDT were similar. Based on this metric, Model 1 was best suited for *β*-HCH, Model 2 for HCB, Trans-nonachlor and *p,p*’-DDE, and Model 3 for *p,p*’-DDT. The DIC and RMSE values were smallest for Model 3 indicating that the most complex model had the best fit for all OCPs both with and without adjustment to the number of variables. The standard deviation of the posterior mean was also smallest for Model 3.

**Table 3:** Summary of model diagnostics of the three hierarchical models, for HCB, *β*-HCH, Trans-nonachlor, *p,p’*-DDE and *p,p’*-DDT by 80%, 90% and 95% predictive intervals (PI), deviance information criteria (DIC), root mean squared error (RMSE) and standard deviation (*σ*) of the posterior mean (Post. mean) with a 95% credible interval. Percentages closer to their respective PIs and lower DIC, RMSE and *σ* values indicate model adequacy.

OCP	Model	80% PI	90% PI	95% PI	DIC	RMSE	*σ* Post. mean (95% CrI)
**HCB**	**Model 1**	76.62	82.47	87.01	219.75	0.67	0.5 (0.44,0.56)
	**Model 2**	86.36	91.56	94.16	212.22	0.46	0.48 (0.43,0.55)
	**Model 3**	96.10	96.10	96.10	141.37	0.35	0.38 (0.34,0.44)
***β*-HCH**	**Model 1**	83.12	90.91	94.16	363.18	0.89	0.79 (0.7,0.9)
	**Model 2**	87.01	92.86	94.81	381.96	0.81	0.84 (0.75,0.94)
	**Model 3**	88.31	94.81	96.75	358.50	0.72	0.78 (0.69,0.88)
**Trans-nonachlor**	**Model 1**	79.87	81.82	83.77	180.65	0.60	0.44 (0.39,0.5)
	**Model 2**	83.12	93.51	96.75	272.66	0.57	0.59 (0.53,0.66)
	**Model 3**	92.21	92.86	94.16	178.30	0.40	0.43 (0.38,0.49)
***p,p*** **’-DDE**	**Model 1**	68.83	77.92	83.12	56.42	0.44	0.29 (0.26,0.33)
	**Model 2**	85.71	92.21	97.40	121.72	0.35	0.36 (0.32,0.41)
	**Model 3**	88.31	96.10	97.40	29.40	0.24	0.27 (0.24,0.3)
***p,p*** **’-DDT**	**Model 1**	85.71	92.21	96.10	338.66	0.74	0.73 (0.65,0.83)
	**Model 2**	88.31	90.26	95.45	336.22	0.70	0.73 (0.65,0.81)
	**Model 3**	81.82	91.56	95.45	331.20	0.67	0.71 (0.63,0.8)

### Variable importance

The relative importance of variables and factors was consistent for all OCPs while the relative contribution of interactions differed between OCPs.

**Fig 2:**
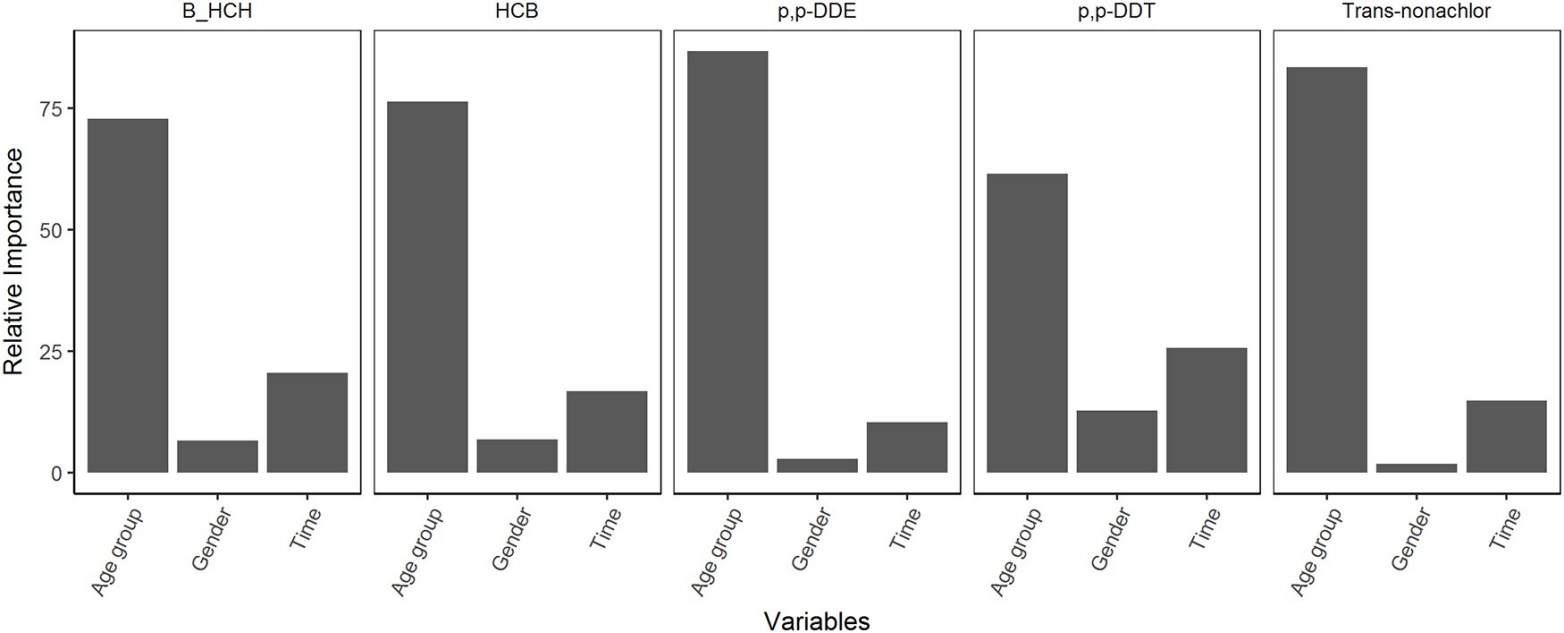
Graphical summary of relative importance of variables in BRT models. Relative importance of variables in predicting the concentrations of *β*-HCH, HCB, *p,p’*-DDE, *p,p’*-DDT and Trans-nonachlor based on a BRT analysis. In order of relative importance (left to right), the variables are age group, time and gender.

**Fig 3:**
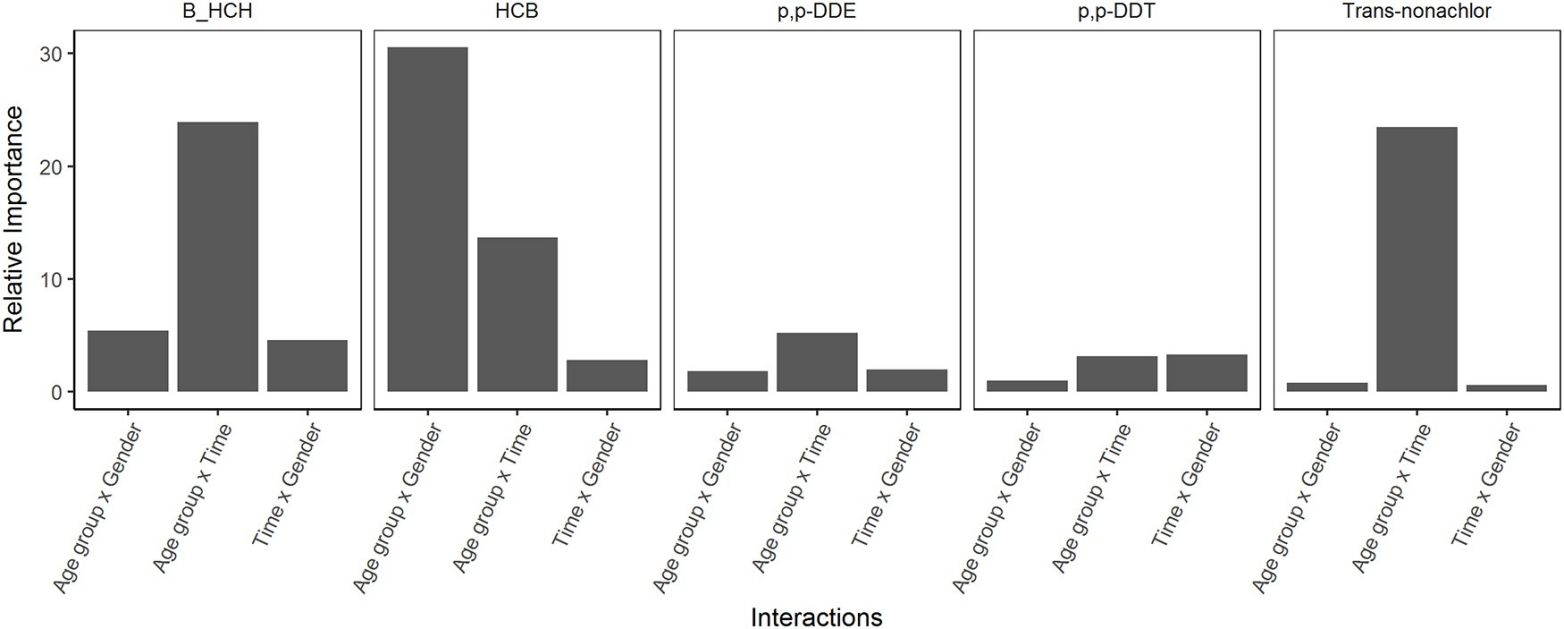
Graphical summary of relative importance of interactions in BRT models. Relative importance of interactions in predicting the concentrations of *β*-HCH, HCB, *p,p’*-DDE, *p,p’*-DDT and Trans-nonachlor based on a BRT analysis. The interactions from left to right are between age group and time, age group and gender, and time and gender.

Results of the BRT analysis (Fig 2) showed that the variable with the highest relative importance was age group followed by time and gender for all OCPs. The importance of age group in the analysis reflected the known persistence and accumulation of OCPs in the body [24, 44]. The relative importance scores for gender was lowest among the predictors. The tree analysis also described the relative importance of interactions between the variables (Fig 3). Age group x gender had the largest relative importance among interaction terms for HCB concentrations; age group x time was the most important interaction term for *β*-HCH, Trans-nonachlor, *p,p’*-DDE and *p,p’* -DDT concentrations. These terms were later fed into the hierarchical model.

**Fig 4:**
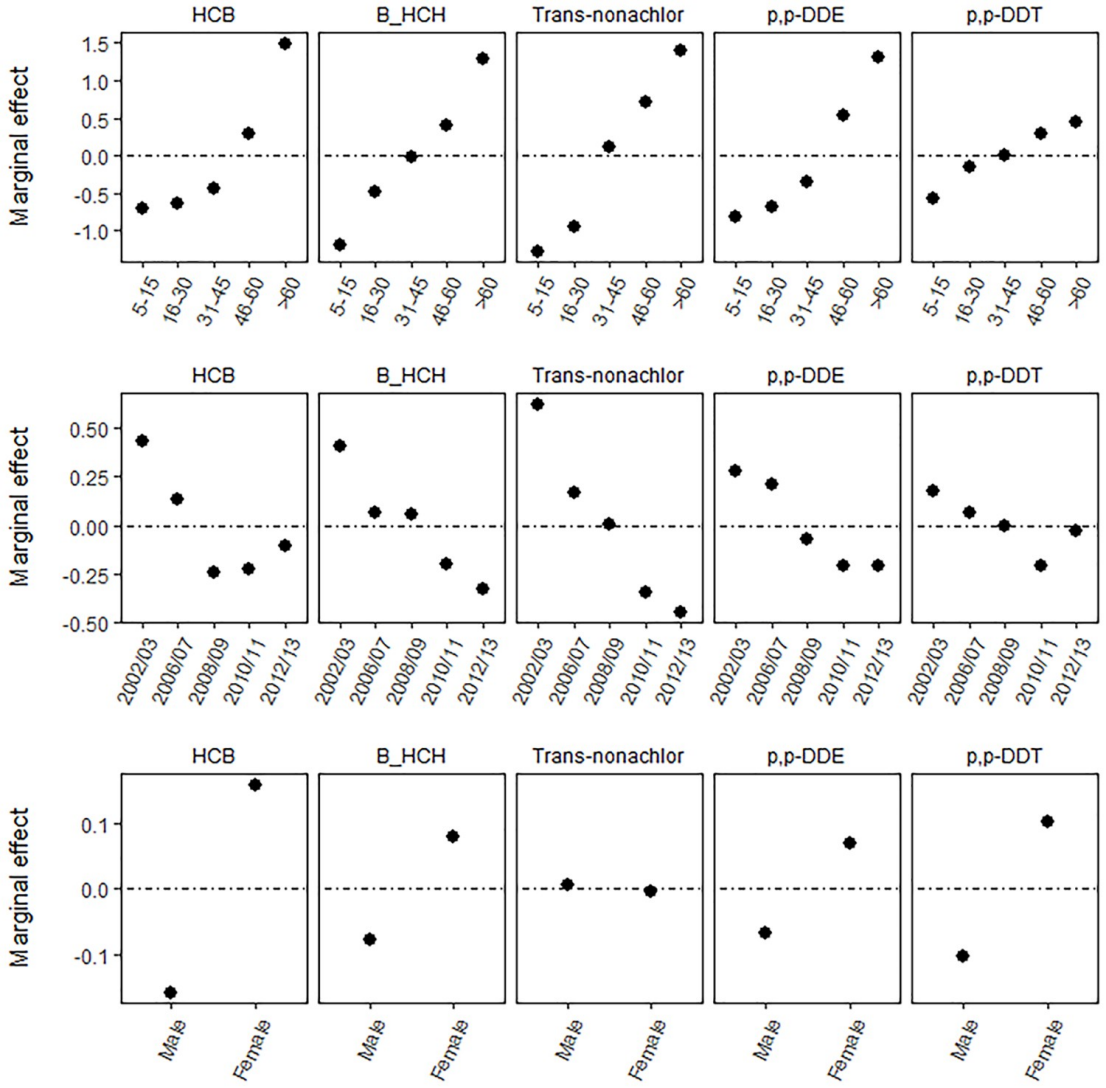
Partial dependence plots of BRT models. Partial dependence plots of the marginal effects of age group (top), time (middle) and gender (bottom) on the concentrations of HCB, *β*-HCH, Trans-nonachlor, *p,p’*-DDE and *p,p’*-DDT where the zero-point of the marginal effect indicates the model expected OCP concentration. Values above zero indicate higher concentrations than expected and values below zero indicate lower concentrations.

Factors that strongly influenced the concentration of HCB and *p,p’*-DDE included the older age groups 46-60 and >60 years, the earlier years 2002/03 and 2006/07 and gender (Fig 4). *β*-HCH and *p,p’* -DDT concentrations were similarly impacted by children aged 0-4 years as well as adults aged 46-60 and >60 years, the years 2002/03, 2006/07 and 2008/09 and gender. The factors that influenced the concentration of Trans-nonachlor included adults aged 31-45, 46-60 and >60 years, the years 2002/03, 2006/07 and 2008/09 and gender.

### Interaction effect on OCP concentrations

Hierarchical models demonstrated evidence of interaction effects between age group and time for *β*-HCH concentrations as well as age group and gender for HCB concentrations. Older age groups were also found to have higher HCB, Trans-nonchlor and *p,p*’-DDE concentrations than their younger counterparts.

**Fig 5:**
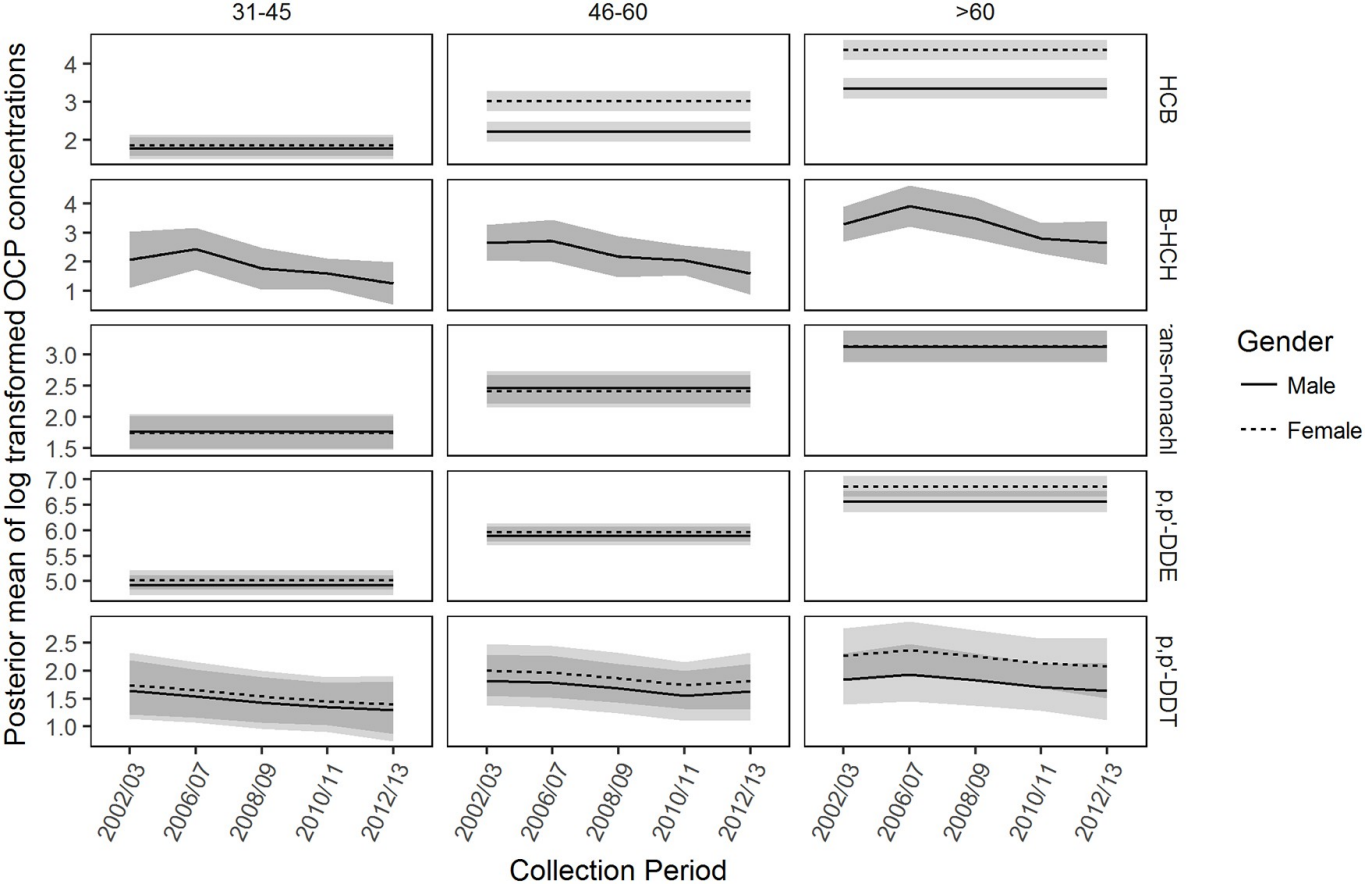
Posterior mean OCP concentrations. Posterior mean log-transformed concentrations of HCB, *β*-HCH, Trans-nonachlor, *p,p’*-DDE and *p,p’*-DDT plotted over the years 2002/03 to 2012/13 for age groups 31-45, 46-60 and >60 year olds. HCB, Trans-nonachlor and *p,p*’-DDE were specified by Model 2, *p,p*’-DDT by Model 3 and *β*-HCH by Model 1. Line type indicates the gender, as described in the figure legend. The light grey bands around the posterior means indicate the 95% credible interval and the dark grey bands indicate an overlap of the 95% credible intervals where there is not enough evidence for the OCP concentration. Units of OCP concentrations were in ng/g lipid.

Results of the hierarchical model are illustrated in Fig 5. There was strong evidence of an interaction effect between age group and time in *β*-HCH concentrations for adults aged >60 years; this age group had higher concentrations compared to children aged <17 years across all years of sample collection. The effect of an interaction between age group and gender was strongly supported in HCB concentrations for adults aged >45 years where women had higher concentrations than men; there was no evidence of the interaction for Trans-nonachlor and *p.p*’-DDE. The concentration of HCB in adults aged >60 years was higher than other age groups. The *p,p*’-DDE concentrations were higher in adults aged >60 years compared to 45-60 years and both age groups had higher concentrations than the younger age groups. The concentrations of Trans-nonachlor were higher in >60, 45-60 and 31-45 year olds compared to the younger age groups. There was no strong support for interaction effects between age group and time as well as age group and gender on *p,p*’-DDT concentrations.

Results of the sensitivity assessment with respect to the age group 5-15 years sampled in 2006/07 showed minimal impact on the posterior mean estimates. Therefore the discrepancy in the number of pooled samples did not distort the modelling results of the analysis. Results can be found in the Supporting Information (S2 Fig).

### Changes in OCP concentrations

**Fig 6:**
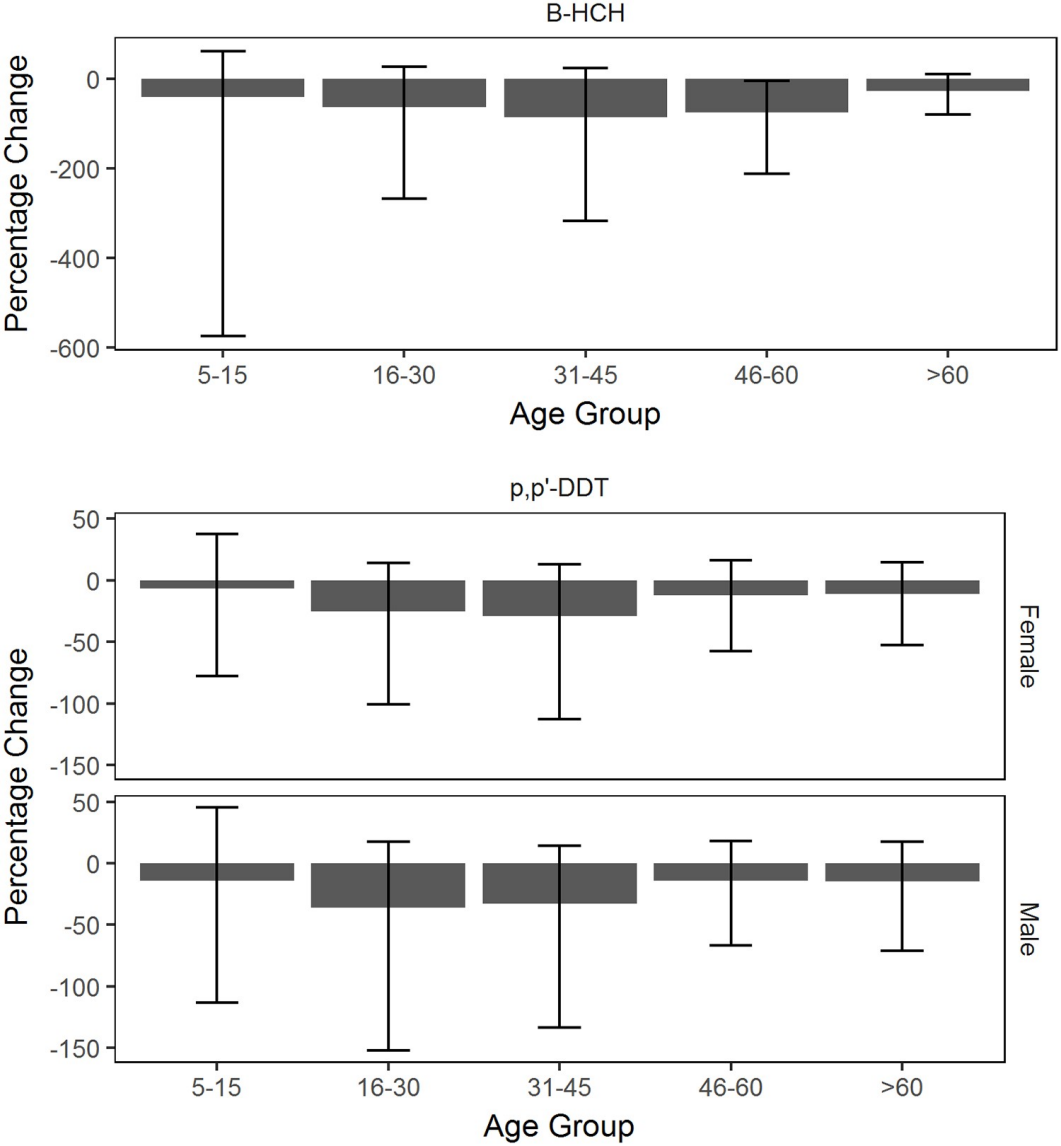
Percent change in OCP concentrations between 2002/03 and 2012/13. The percent change (%) of *β*-HCH and *p,p’*-DDT concentrations (ng/g lipid) between 2002/03 and 2012/13 for age groups 5-15, 16-30, 31-45, 46-60 and >60. The error bars for each age group indicate the 95% credible interval of the percent change.

**Fig 7:**
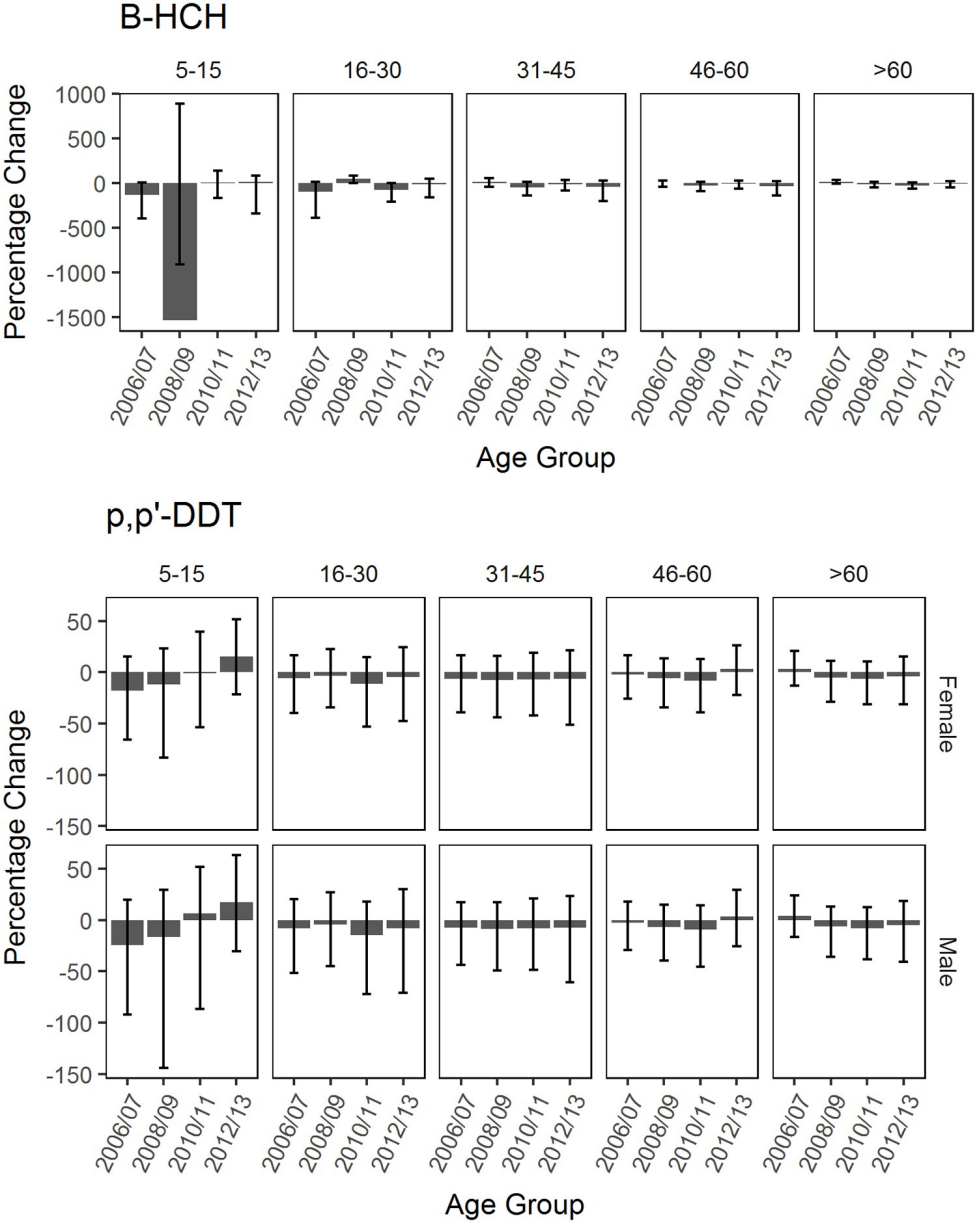
Percent change in OCP concentrations between consecutive sampling times. The percent change (%) of *β*-HCH and *p,p’*-DDT concentrations (ng/g lipid) between each consecutive sampling times for age groups 5-15, 16-30, 31-45, 46-60 and >60. For each age group, the sampling times listed from left to right indicate the differences between 2002/03 and 2006/07, 2006/07 and 2008/09, 2008/09 and 2010/11, and 2010/11 and 2012/13. The error bars for each age group indicate the 95% credible interval of the percent change.

**Fig 8:**
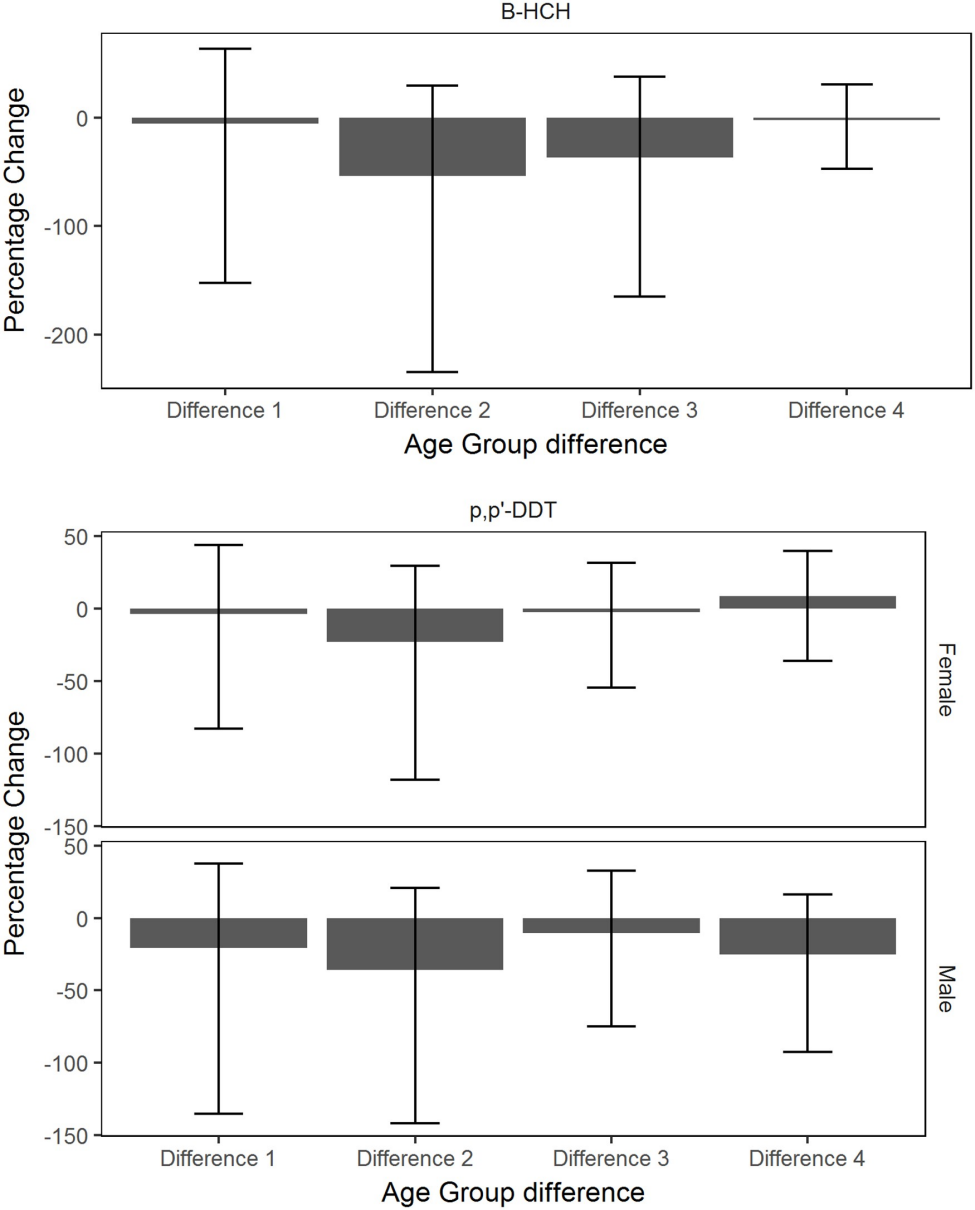
Percent change in OCP concentrations as sample population ages. The percent change (%) of *β*-HCH and *p,p’*-DDT concentrations (ng/g lipid) between an age group sampled in 2002/03 and its adjacent older age group sampled in 2012/13. The error bars for each age group indicate the 95% credible interval of the percent change. L-R: Difference 1: 5-15 year olds in 2002/03 and 16-30 year olds in 2012/13, Difference 2: 16-30 year olds in 2002/03 and 31-45 year olds in 2012/13, Difference 3: 31-45 year olds in 2002/03 and 45-60 year olds in 2012/13, Difference 4: 45-60 year olds in 2002/03 and >60 year olds in 2012/13.

All age groups for *β*-HCH and *p,p*’-DDT showed a decrease in concentrations between 2002/03 and 2012/13 (Fig 6). There was strong evidence of a decrease in *β*-HCH concentration between 2002/03 and 2012/13 for adults aged 45-60 years. There were no distinct changes in *β*-HCH and *p,p*’-DDT concentrations between each consecutive time of sample collection (Fig 7). There was no strong evidence of a change in *β*-HCH and *p,p*’-DDT concentrations between an age group in 2002/03 and its extrapolated age group in 2012/13 (Fig 8). As time was not specified in Model 2, HCB, Trans-nonachlor and *p,p*’-DDE were excluded from this analysis. The interpreted effect of age on the concentrations of HCB, Trans-nonachlor and *p,p*’-DDE could also be related to birth cohort effects, taking into account the historical use of OCPs prior to 1985.

## Discussion

This paper has proposed a two-stage analysis for the exploration of age group, gender and temporal effects, and their interaction, on OCP concentrations. Using BRTs and hierarchical modelling, three statistical inferences were presented, using data on serum OCP concentrations. These inferences focused on the relative importance of variables and interactions, the effects of interactions on OCP concentrations and the change in OCP concentrations over time. The inferences highlighted strong evidence for an interaction effect between age group and time for *β*-HCH concentrations in adults aged >60 years as well as age group and gender for HCB concentrations in adults aged >45 years.

The application of BRTs for identifying key interactions was motivated by its underlying non-parametric approach that allowed the tree to model non-linear associations as well as the model’s robustness to outliers and missing data [32, 36, 45]. Information on the relative importance of all possible interactions, without needing to specify these terms prior to model implementation, is a key benefit of BRTs. Important interaction terms from the BRT were utilised in a hierarchical model which allowed multiple levels of the data to be included in a single model [46].

Previous analyses of OCP concentrations over multiple years have demonstrated a decline in OCP concentrations over time [18, 19, 47, 48]. A major cause of the declining trend has been attributed to the ban of the commercial or agricultural use of OCPs in various countries [18, 19].

In this paper, there was a similar negative trend for both *β*-HCH and *p,p*’-DDT concentrations, but the analysis showed no strong evidence that there was a substantive decrease in these OCP concentrations. This is not unexpected, since the study period spans years, long after major historical emissions [8] and the smaller ongoing emissions during the study period may result in lower and more diffuse exposure in humans, based on latent pathways into human serum [18, 49, 50].

In studies where OCP concentrations were sampled from various age groups, lower concentration of chemicals have been found in younger age groups [27, 51–53]. The same trend was observed in this study with the two-stage analysis providing strong evidence for larger OCP concentrations in older age groups. The difference in concentrations between age groups has been attributed to the implementation of bans on OCPs; older age groups had a longer exposure history to OCPs as they had lived through the agricultural use of the chemicals and hence had accumulated higher concentrations [54, 55] compared to their younger counterparts.

A number of studies had also found differences in OCP concentrations between genders; some studies found higher concentrations in women [26, 56, 57] while other studies found higher concentrations in men [27, 58, 59]. In the current study, the analysis showed higher concentrations of HCB, *β*-HCH, *p,p*’-DDE and *p,p*’-DDT in women, and strong evidence of this in HCB concentrations. Hypotheses on why there is a difference in concentrations between genders differ depending on age of the females. For parous (corresponding to our 15-30 and 31-45 year olds) females there is excretion due to placental transfer and breastfeeding [60]. In older females, this factor as discussed by Salihovic et al. (2012) is no longer relevant as the time since breastfeeding has long since past. While Salihovic et al. [59] suggest that females have higher body fat than males, Porta et al. [26] investigated OCPs and BMI and could not explain the higher concentrations in older females compared to males. Further work is required on gender differences in regard to OCP concentrations if there are likely to be any clinical repercussions.

Further to the main effects of age group, gender and time, most studies on OCP have not analysed the effect of interactions on the concentrations [21]. Thomas et al. [22] found that the interaction between age group and time had a significant effect on the HCB concentration and was best implemented for modelling *β*-HCH and *p,p*’-DDT. Thomas et al. [22] also found a significant interaction effect between age group and gender on *p,p*’-DDT concentrations. In this study, HCB, Trans-nonachlor and *p,p*’-DDE concentrations were best modelled by the interaction between age group and gender, without a temporal effect. This suggested that there was negligible change in OCP concentrations over the times of sample collection that were studied. This is possibly related to the indirect pathways of HCB, Trans-nonachlor and *p,p*’-DDE [18, 49, 50] into human serum long after its historical use [8]. Gender did not have a strong effect on the *β*-HCH concentrations which were modelled as a function of the interaction between age group and time. *p,p*’-DDT concentrations were modelled by interactions between age group and gender as well age group and time demonstrating the effect of age group, gender and time on the change in concentrations.

### Limitations

Results did not provide a complete overview of temporal trends across all age groups as no data was collected for the years 2002/03 for 0-4 year olds, and 2004/05 for all age groups.

The analysis was unable to account for confounding factors such as metabolism rate, diet, place of residence and weight loss due to the pooled sampling method adopted to collect data.

### Extensions

The hierarchical model utilised in this paper could be further extended to incorporate prior knowledge, elicited from experts or meta-analyses of published studies. It may be more appropriate for prior information to come from experts in the field as there are no standardised methods for laboratory analysis, detection limits, number of people sampled, age ranges, years sampled, number of years sampled and whether samples were pooled to lower laboratory costs [26, 61]. Prior information can be included as information on the coefficient values for the interactions between age group and gender, and age group and time.

In the current study, each OCP was modelled individually. This is useful as each chemical has a different history of production and use. However, OCPs could be modelled in a multivariate analysis that accounts for any positive correlations between OCPs such as that between *p,p*’-DDT and *p,p*’-DDE.

The model can also be applied to other POPs such as perfluorinated chemicals and polybrominated diphenyl ethers in order to investigate the effects of age group, gender and time as well as any interactions between these variables.

## Supporting information

S1 Fig Graphical summary of Gelman-Rubin diagnostic for Models 1, 2 and 3 of HCB, *β*-HCH, Trans-nonachlor, *p,p*’-DDE and *p,p*’-DDT to demonstrate model convergence. Point estimates and 95% confidence intervals of potential scale reduction factors (Y axis) are plotted for each parameter of the model (X axis). Estimates close to 1 indicate chain convergence for the model parameter.
(TIF)

S2 Fig Comparison of the posterior mean of Models 1, 2 and 3 for HCB, *β*-HCH, Trans-nonachlor, *p,p*’-DDE and *p,p*’-DDT concentrations for males and females in the age group 5-15 sampled in 2006/07 using two datasets. The first dataset (represented in red) comprised of the original number of samples (15 concentration samples of males and 17 concentration samples of female) for the sub-population and the second dataset (represented in blue) consisted of four randomly sampled concentrations for each gender of the sub-population to match the number of samples for the remaining age groups. The posterior mean under each model was defined as: *β*_*at*_ for Model 1, *α*_*ag*_ for Model 2 and *β*_*at*_ + *α*_*ag*_ for Model 3. The analysis demonstrated no strong evidence of a difference in the posterior mean estimate between the original and randomly sampled data for any OCP under any model.
(TIF)

S1 File Details of sample collection and analysis. (PDF)
