# Supplementary figures and images for: Application of ensemble methods to analyse the decline of organochlorine pesticides in relation to the interactions between age, gender and time
Source: PLoS One. 2019 Nov 13;14(11):e0223956. doi: 10.1371/journal.pone.0223956 (PMC6853321; doi:10.1371/journal.pone.0223956)

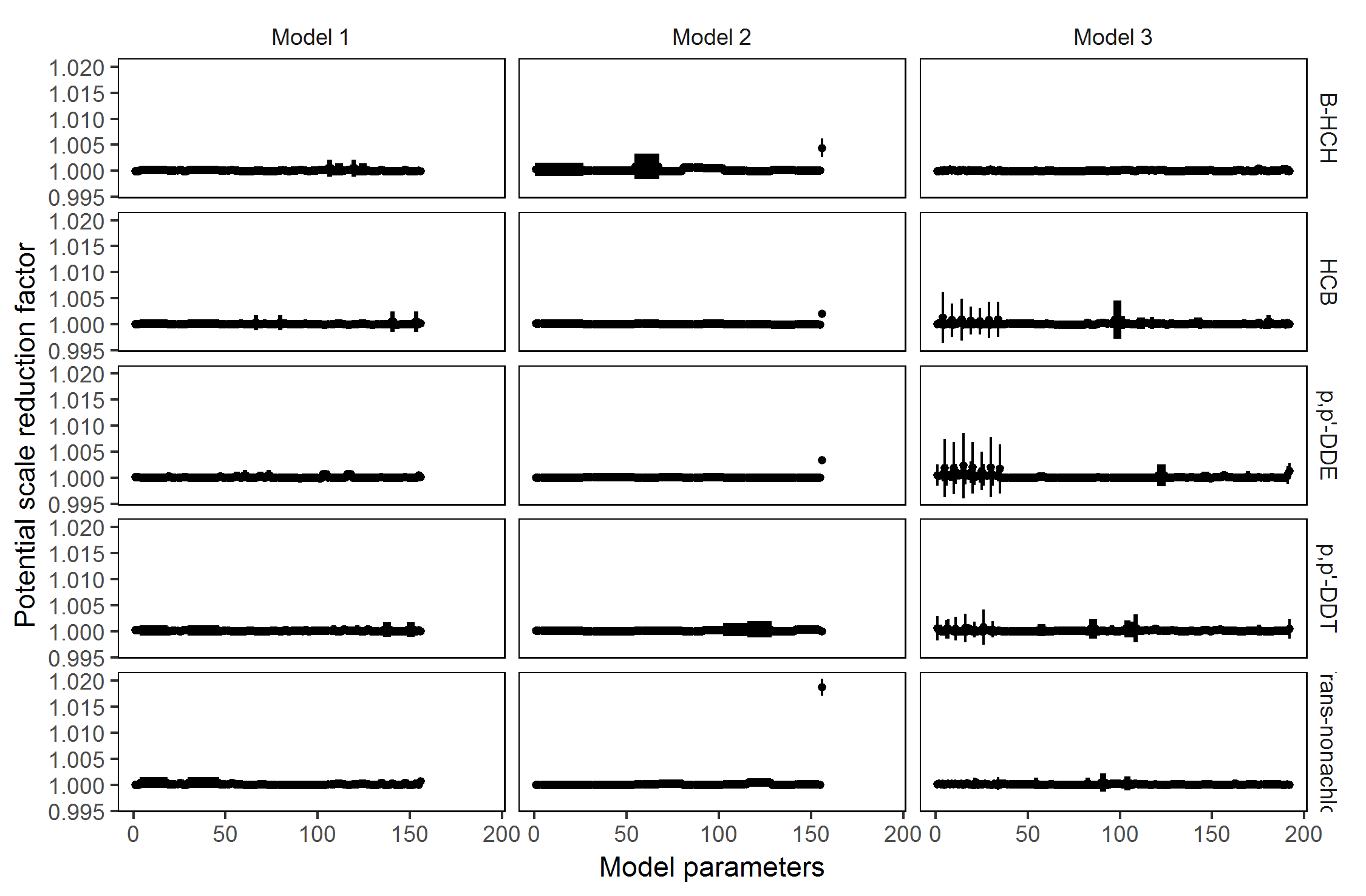

Supplement: S1 Fig — Point estimates and 95% confidence intervals of potential scale reduction factors (Y axis) are plotted for each parameter of the model (X axis). Estimates close to 1 indicate chain convergence for the model parameter. (TIF) [file pone.0223956.s001.tif]

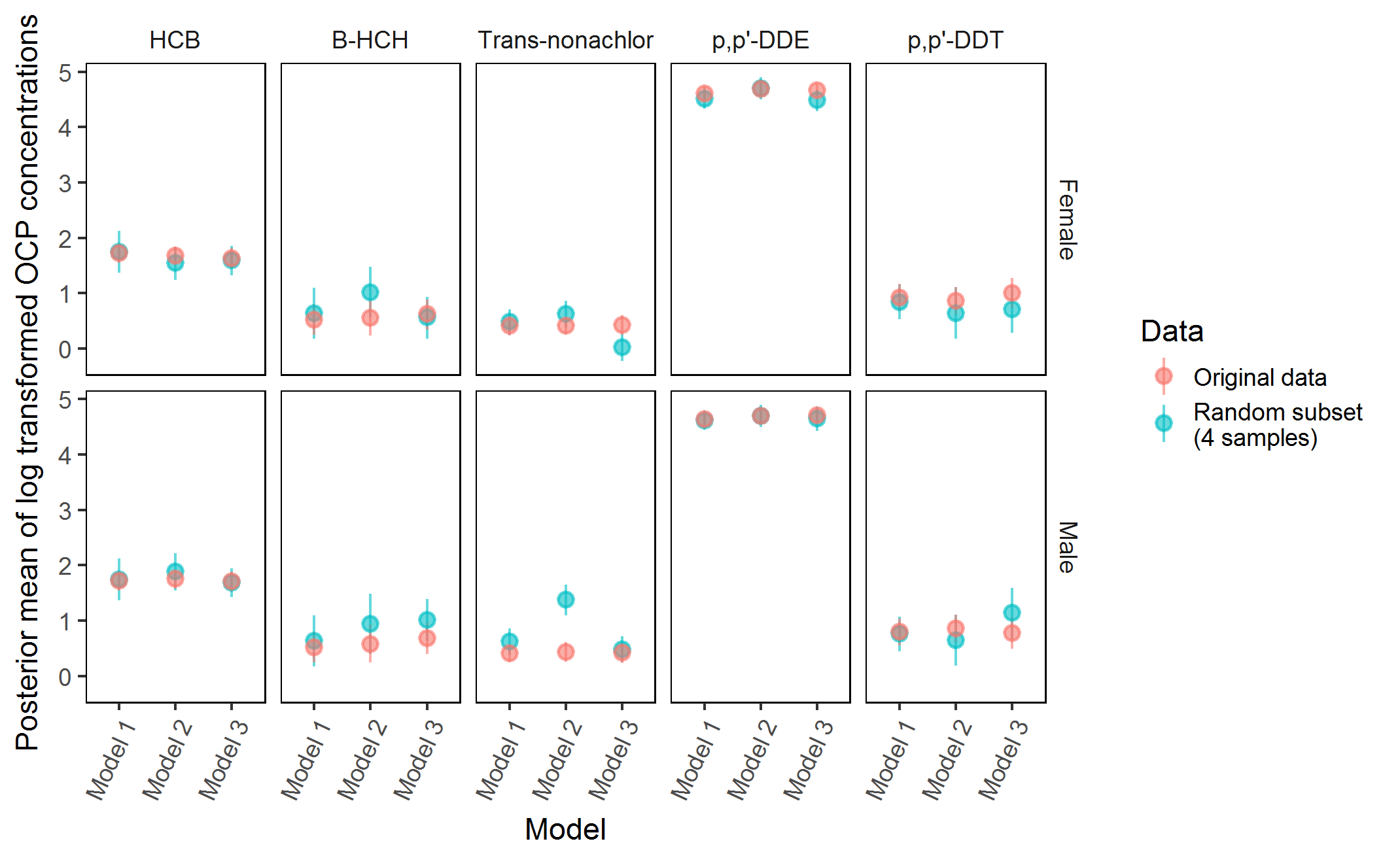

Supplement: S2 Fig — The first dataset (represented in red) comprised of the original number of samples (15 concentration samples of males and 17 concentration samples of female) for the sub-population and the second dataset (represented in blue) consisted of four randomly sampled concentrations for each gender of the sub-population to match the number of samples for the remaining age groups. The posterior mean under each model was defined as: βat for Model 1, αag for Model 2 and βat + αag for Model 3. The analysis demonstrated no strong evidence of a difference in the posterior mean estimate between the original and randomly sampled data for any OCP under any model. (TIF) [file pone.0223956.s002.tif]
