# Supplementary material for: Application of ensemble methods to analyse the decline of organochlorine pesticides in relation to the interactions between age, gender and time
Source: PLoS One. 2019 Nov 13;14(11):e0223956. doi: 10.1371/journal.pone.0223956 (PMC6853321; doi:10.1371/journal.pone.0223956)
Supplement: S1 File — (PDF) [file pone.0223956.s003.pdf]

Sample Collection

Pooled samples of human blood serum from males and females were collected in metropolitan South East Queensland, Australia in 2002/03, 2006/07, 2008/09, 2010/11 and 2012/13. These samples were used to provide robust serum data on OCP concentrations for five time points over the past decade. All samples were obtained in collaboration with Sullivan Nicolaides Pathology (SNP) from de-identified surplus pathology samples. Stratification criteria included age (Table 1) and gender.

Table 1: Stratification of age (years) for each collection year.

2002/03	2006/07	2008/09	2010/11	2012/13
**-**	Umbilical Cord sample	-	-	-
**-**	0-0.5	-	-	-
**-**	0.6-1	-	-	-
**-**	1.1-1.5	-	-	-
**-**	1.6-2	-	-	-
**-**	2.1-2.5	-	-	-
**-**	2.6-3	-	-	-
**-**	3.1-3.5	-	-	-
**-**	3.6-4	-	-	-
**-**	-	0-4	0-4	0-4
**-**	4.1-6	-	-	-
**-**	6.1-9	-	-	-
**-**	9.1-12	-	-	-
**-**	12.1-15	-	-	-
**-**	5-15	5-15	5-15	5-15
**<16**	-	-	-	-
**16-30**	16-30	16-30	16-30	16-30
**31-45**	31-45	31-45	31-45	31-45
**46-60**	46-60	46-60	46-60	46-60
**>60**	>60	>60	>60	>60

Each serum pool comprised 100 individual de-identified surplus serum samples, except those pools created for the 2006/07 sampling that contained up to 30 samples per pool. A replicate pool was created for each strata (age group, gender and collection period). Specific details on the sampling regime is given in [1–3].

In total, 12,175 individual samples were used to make 183 pools; 26 pools (n = 2555) in 2002/03, 85 pools (n = 2420) in 2006/07, and thereafter 24 pools (n = 2400) were used (2008/09 until 2012/13). While within a sampling period no donor contributed more than one sample to the study, it was not possible to determine if any one donor contributed to more than one collection period. University of Queensland Medical Research Ethics Committee and the Queensland University of Technology Ethics Committee granted ethics approval for this study.

Sample Analysis

The OCPs investigated were: hexachlorobenzene (HCB), β -hexachlorocyclohexane (β-HCH), γ -hexachlorocyclohexane (lindane) (γ-HCH), oxy-chlordane, trans-nonachlor, *p,p*'-DDE, o,p'-DDT, *p,p*'-DDT and Mirex. The samples were analyzed using the methods that have been described previously [4]. Briefly, a set of samples was defined as 24 unknown samples with three analytical blanks and three

quality control/quality assurance (QC/QA) samples and were processed using a semi-automated sample preparation method. Human sera (2 g) were weighed into test tubes and fortified with internal standards (13C-labeled) using a 215 Liquid Handler (Gilson Inc, Middleton, WI). Then, formic acid and water were added to denature proteins and dilute the samples on the liquid handler. The target analytes were extracted into dichloromethane using the solid phase extraction (SPE) workstation (Rapid Trace®, Zymark, Hopkinton, MA). Clean up was performed on a two layered column. The top layer comprised activated silica and the bottom layer comprised silica gel/sulfuric acid (2:1 by weight). The top layer retained polar lipids such as cholesterol, while the bottom layer degraded the remaining lipids to produce an extract suitable for the measurement of target analytes. This procedure was automated using the modular SPE workstation. Samples were evaporated to 1 mL and transferred to the gas chromatograph vials, which were previously spiked with recovery standards. Samples were further evaporated to 10 L and analyzed by gas chromatography high resolution mass spectrometry. A DFS (ThermoFinnigan, Bremen, Germany) instrument was used for the analysis. The chromatographic separations were carried out on a 6890 gas chromatograph (Agilent Technologies, Atlanta, GA) fitted with a DB5HT capillary column [(15m, 0.25mm inner diameter, and 0.10 m thickness)]. The results are expressed as ng/g lipid and are reported to two significant figures. Where a chemical was found to be below the limit of detection (LOD) it is reported as <LOD. The LOD was dependent on sample size and blanks. The samples were analyzed in three runs; the 2006/07 samples were analyzed in 2007, the 2002/03 and 2008/09 samples were analyzed in 2009 and the 2010/11 and 2012/13 samples were analyzed in 2013.

Quality Assurance / Quality Control (QA/QC)

Six blind field blanks were included in the analytical runs (two in the 2006/07 analysis; one each in the 2008/09, 2010/11 and 2012/13 analyses). The field blanks were comprised of bovine serum (Sigma Aldrich B8655), expected to have OCP concentrations below the detection limit in the analytical methodology. The blanks used were aliquoted into the collection tubes used by the pathology laboratory, frozen, defrosted and placed in the sampling containers used for human samples. No OCPs were detected in the blanks, indicating that contamination did not occur during the sample collection and pooling process.

References

[1] Harden F, Toms L, Päpke O, Ryan J, Müller J. Evaluation of age, gender and regional concentration differences for dioxin-like chemicals in the Australian population. Chemosphere. 2007;67(9):S318–S324.

[2] Toms L, Sjödin A, Harden F, Hobson P, Jones R, Edenfield E, et al. Concentrations Of Polybrominated Diphenyl Ethers (PBDEs) In Pooled Human Serum Are Higher In Children (aged 2-5 Years) Than In Infants And Adults. Environmental Health Perspective. 2009;117:1461–1465.

[3] Toms LM, Harden F, Hobson P, Sjödin A, Mueller J. Temporal trend of organochlorine pesticides in Australia. Organohalogen Compounds. 2012;74: 775–778.

[4] Sjödin A, Jones RS, Lapeza CR, Focant JF, McGahee EE, Patterson DG. Semiautomated high-throughput extraction and cleanup method for the

measurement of polybrominated diphenyl ethers, polybrominated biphenyls, and polychlorinated biphenyls in human serum. Analytical chemistry. 2004;76(7):1921–1927.
