# Supplementary material for: Application of ensemble methods to analyse the decline of organochlorine pesticides in relation to the interactions between age, gender and time
Source: PLoS One. 2019 Nov 13;14(11):e0223956. doi: 10.1371/journal.pone.0223956 (PMC6853321; doi:10.1371/journal.pone.0223956)
Supplement: Attachment — Submitted filename: response_280519.pdf [file pone.0223956.s004.pdf]

Response to editor's comments

PONE-D-17-12878R2

Application of ensemble methods to analyse the decline of organochlorine pesticides in relation to the interactions between age, gender and time

We thank the editor for the opportunity to respond to the points outlined in the decision dated September 06, 2018. As detailed in an email on 26th May 2019, the first author for this manuscript has taken up employment elsewhere and the contact address for the submission (aleysha.thomas@qut.edu.au) is no longer valid. As coauthors, we have only just become aware that this decision had been communicated, hence the very long delay in our reply.

Editor's comment
I have concerns about readability of a paragraph in the revised discussion. I therefore request that you revise the text again to improve the overall readability of the discussion.

Minor comment:
Lines 291-296:
"The same trend was replicated in this paper for β-HCH and p,p'-DDT concentrations, however the analysis demonstrated no strong evidence for the decrease in these OCP concentrations. Although the replicated trend was expected, the lack of evidence for a decrease in concentrations reflects the historical use of the chemicals [8] and latent pathways into human serum [18, 49, 50], possibly leading to smaller doses."

As a reader, I would be confused by these sentences. The first part of the first sentence states a trend (consider rephrasing 'the same' as it is probably not the exact 'same') but the second part states that there was no strong evidence for the decrease and is inconsistent with the first part. Please clarify what you would like to emphasize (directionality and magnitude of trend?).
The second sentence writes that the lack of evidence for a decrease reflects historical use of the chemicals. Do you rather mean that the study period spans years long after major historical emissions and that the minimal ongoing emissions during the study period results in low and diffuse exposure to humans and thus lower concentrations are expected?

Please rephrase these sentences.

Response
We acknowledge that these sentences are not sufficiently clear and we have revised them as follows.

In this paper, there was a similar negative trend for both β-HCH and p,p'-DDT concentrations, but the analysis showed no strong evidence that there was a substantive decrease in these OCP concentrations. This is not unexpected, since the study period spans years, long after major historical emissions [8], and the smaller ongoing emissions during the study period may result in lower and more diffuse exposure in humans, based on latent pathways into human serum [18, 49, 50].
