# Supplementary material for: Application of ensemble methods to analyse the decline of organochlorine pesticides in relation to the interactions between age, gender and time
Source: PLoS One. 2019 Nov 13;14(11):e0223956. doi: 10.1371/journal.pone.0223956 (PMC6853321; doi:10.1371/journal.pone.0223956)
Supplement: Peer review history

# Author comment: Author response to previous submission

**DOI:** 10.1371/journal.pone.0223956.r001

5 Jun 2019

Attachment Submitted filename: response_280519.pdf Click here for additional data file.

# Editor report: Decision Letter 0

**DOI:** 10.1371/journal.pone.0223956.r002

**Authors:** Timothy J Garrett

3 Oct 2019

Application of ensemble methods to analyse the decline of

organochlorine pesticides in relation to the interactions

between age, gender and time

PONE-D-19-15919

Dear Dr. Thomas,

We are pleased to inform you that your manuscript has been judged scientifically suitable for publication and will be formally accepted for publication once it complies with all outstanding technical requirements.

Within one week, you will receive an e-mail containing information on the amendments required prior to publication. When all required modifications have been addressed, you will receive a formal acceptance letter and your manuscript will proceed to our production department and be scheduled for publication.

Shortly after the formal acceptance letter is sent, an invoice for payment will follow. To ensure an efficient production and billing process, please log into Editorial Manager at https://www.editorialmanager.com/pone/, click the "Update My Information" link at the top of the page, and update your user information. If you have any billing related questions, please contact our Author Billing department directly at authorbilling@plos.org.

If your institution or institutions have a press office, please notify them about your upcoming paper to enable them to help maximize its impact. If they will be preparing press materials for this manuscript, you must inform our press team as soon as possible and no later than 48 hours after receiving the formal acceptance. Your manuscript will remain under strict press embargo until 2 pm Eastern Time on the date of publication. For more information, please contact onepress@plos.org.

With kind regards,

Timothy J Garrett, PhD

Academic Editor

PLOS ONE

1. Thank you for stating the following financial disclosure:

"The funders had no role in study design, data collection and analysis, decision to publish, or preparation of the manuscript."

1. Please provide an amended Funding Statement that declares *all* the funding or sources of support received during this specific study (whether external or internal to your organization) as detailed online in our guide for authors at http://journals.plos.org/plosone/s/submit-now.  

1. Please state what role the funders took in the study.  If any authors received a salary from any of your funders, please state which authors and which funder. If the funders had no role, please state: "The funders had no role in study design, data collection and analysis, decision to publish, or preparation of the manuscript."

Please send your amended statements by return email; we will change the online submission form on your behalf.

Additional Editor Comments (optional):

I'm sorry for the length of time for review. I had several reviewers not able to review this article. In the end, I decided to review the manuscript based and make an assessment based on the original review.  I realize the circumstances regarding the email chain that was lost regarding that review.  Your responses to the initial comments are acceptable.  Your article is well-written with good background detail on the methods used. 

Reviewers' comments:

# Editor report: Acceptance letter

**DOI:** 10.1371/journal.pone.0223956.r003

**Authors:** Timothy J Garrett

1 Nov 2019

PONE-D-19-15919 

Application of ensemble methods to analyse the decline of organochlorine pesticides in relation to the interactions between age, gender and time 

Dear Dr. Thomas:

I am pleased to inform you that your manuscript has been deemed suitable for publication in PLOS ONE. Congratulations! Your manuscript is now with our production department. 

If your institution or institutions have a press office, please notify them about your upcoming paper at this point, to enable them to help maximize its impact. If they will be preparing press materials for this manuscript, please inform our press team within the next 48 hours. Your manuscript will remain under strict press embargo until 2 pm Eastern Time on the date of publication. For more information please contact onepress@plos.org.

For any other questions or concerns, please email plosone@plos.org. 

Thank you for submitting your work to PLOS ONE.

With kind regards,

PLOS ONE Editorial Office Staff

on behalf of

Dr. Timothy J Garrett 

Academic Editor

PLOS ONE
